# Cystatin C, a potential marker for cerebral microvascular compliance, is associated with white-matter hyperintensities progression

**DOI:** 10.1371/journal.pone.0184999

**Published:** 2017-09-14

**Authors:** Woo-Jin Lee, Keun-Hwa Jung, Young Jin Ryu, Jeong-Min Kim, Soon-Tae Lee, Kon Chu, Manho Kim, Sang Kun Lee, Jae-Kyu Roh

**Affiliations:** 1 Department of Neurology, Seoul National University Hospital, Seoul, South Korea; 2 Program in Neuroscience, Neuroscience Research Institute of SNUMRC, College of Medicine, Seoul National University, Seoul, South Korea; 3 Department of Radiology, Seoul National University Hospital, Seoul, South Korea; 4 Department of Neurology, Chung-Ang University Hospital, Seoul, South Korea; 5 Department of Neurology, The Armed Forces Capital Hospital, Sungnam, South Korea; Henry Ford Health System, UNITED STATES

## Abstract

Cerebral white matter hyperintensities (WMHs) are central MRI markers of the brain aging process, but the mechanisms for its progression remain unclear. In this study, we aimed to determine whether the baseline serum cystatin C level represented one mechanism underlying WMH progression, and whether it was associated with the long-term progression of cerebral WMH volume in MRI. 166 consecutive individuals who were ≥50 years of age and who underwent initial/follow-up MRI evaluations within an interval of 34–45 months were included. Serum cystatin C level, glomerular-filtration rate (GFR), and other laboratory parameters were measured at their initial evaluation and at the end of follow-up. Cerebrovascular risk factors, medications, and blood-pressure parameters were also reviewed. WMH progression rate was measured by subtracting WMH volume at baseline from that at the follow-up using volumetric analysis, divided by the MRI intervals. At baseline, WMH volume was 9.61±13.17 mL, mean GFR was 77.3±22.8 mL/min, and mean cystatin C level was 0.92±0.52 mg/L. After 37.9±3.4 months, the change in WMH volume was 3.64±6.85 mL, the progression rate of WMH volume was 1.18±2.28 mL/year, the mean ΔGFR was 2.4±7.9 mL/min, and the mean Δcystatin C was 0.03±0.34 mg/L. The progression rate of WMH volume was linearly associated with cystatin C level (B coefficient = 0.856; 95% confidence interval [CI] 0.174−1.538; *P* = 0.014), along with the baseline WMH volume (B = 0.039; 95% CI 0.019−0.059; *P*<0.001), after adjusting for the conventional vascular risk factors, laboratory parameters, medication profiles, and GFR. Especially, patients with a baseline level of cystatin C ≥1.00 mg/L exhibited a much higher progression rate of WMH as compared with those with a baseline level of cystatin C <1.00 mg/L (1.60±1.91 mL/year vs. 0.82±1.63 mL/year, *P* = 0.010). We concluded that serum cystatin C level is independently associated with the long-term progression rate of the cerebral WMH volume. Therefore, serum cystatin C level might predict the progression of cerebral WMH.

## Introduction

Cerebral white matter hyperintensity (WMH) is a central magnetic resonance imaging (MRI) marker of the brain aging process, and are largely associated with certain neurological diseases including dementia and stroke.[1–3] Numerous studies have indicated that the progression of WMH is due to the chronic impairment of the soluble metabolite clearance from the brain parenchyma via the glymphatic system in the perivascular spaces of cerebral penetrating arterioles.[4–7] As the main motive of the solute clearance via the glymphatic system is the pulsation of cerebral penetrating arterioles,[1,5,6] cerebrovascular stiffness was known to be the major mechanism underlying WMH progression. In this regard, various factors related with vascular compliance, such as advanced age, hypertension,[1,8,9] pulse pressure,[10,11] aortic pulse-wave velocities,[11,12] middle cerebral artery pulsatility index (PI),[13] and glomerular filtration rate (GFR)[14–16] have been regarded as risk factors for WMH. There is evidence that WMH might be attributable to the distinct physiologic properties of the cerebral penetrating arterioles, with a much higher capacity of pulsation than that of the pial vessels, and a gradually reduced pulsation with the increase in age.[4,17] Therefore, a marker that reflects the integrity of the cerebral penetrating arterioles and its solute clearance function is required in order to appropriately predict or intervene in the progression of WMH.

Cystatin C, a cysteine proteinase inhibitor, has been recognized as a marker that measures GFR more precisely than the creatinine-based methods. Given that the cystatin C-based GFR estimation is not affected by muscle mass or race,[18–20] previous cross-sectional studies have reported that serum cystatin C level correlates with WMH severity.[19,21] Moreover, serum cystatin C level might also reflect the functional status of cerebral penetrating arterioles and the activity of neuronal degeneration process, as cystatin C is highly secreted from neurons and glial cells[22–25] and deposited in brain parenchyma and the walls of microvessels, and its accumulation might induce further neuronal and vascular degeneration.[21,22]

In the present study, we hypothesized that serum cystatin C levels might represent one mechanism underlying the progression of cerebral WMH. Thus, we aimed to evaluate whether serum cystatin C level might be associated with the long-term progression of cerebral WMH volume in MRI, independently from the previously established systemic and renal factors associated with WMH.

## Materials and methods

### Study population

In the present retrospective study, the study population was identified from consecutive patients who were ≥50 years of age; who visited a tertiary hospital between January 2005 and March 2012; and who underwent baseline MRI/magnetic resonance angiogram (MRA) scan and laboratory evaluations, including serum cystatin C levels and urine spot microalbumin/creatinine ratio at the baseline and the end of the 34–45-month follow-up period. The 34–45 months between the baseline and follow-up evaluations were designated according to previous studies that have investigated the progression of WMH with a typical follow-up duration of about 3 years.[3,9,17,26–28] To minimize the potential effects on WMH progression, the following exclusion criteria were applied to the 263 initially-included patients: patients had (1) a significant (≥ 30%) stenosis in the intra/extracranial arteries according to initial MRA results; (2) an active systemic illness or an inability to carry out daily activities independently; (3) a history of stroke, but not an old (>90 days) lacunar stroke, and/or major head trauma, brain surgery, intracranial radiation therapy, or other evidence of chronic disorders involving the central nervous system (CNS); and (4) an MRI image of poor quality for evaluation. According to these criteria, 35 patients with significant cerebral arterial stenosis, 10 patients with active systemic illness or who were incapable of independent daily living, and 52 patients with a stroke history other than an old lacunar infarction or who a demonstrated CNS disease were sequentially excluded. Three patients with end-stage renal disease (ESRD, GFR >15 mL/min) were also excluded, as ESRD is known to have a distinct influence on the progression of WMH via the alteration of brain homeostasis.[29] Thus, the remaining 166 individuals qualified for final analysis (**S1 Table, panel A**). The image quality of the baseline and follow-up MRIs were good for analysis in each patient. The baseline MRI evaluation was done as a part of regular medical check-up program provided by Seoul National University Hospital Healthcare System in 100 (60.2%) patients, during the evaluation for headache or dizziness in 37 (22.3%) and for a single small (≤3 mm) unruptured intracranial aneurysm in 10 (6.0%), or in a follow-up evaluation of an old lacunar infarction in 19 (11.4%). Indications for follow-up MRI included a regular medical check-up program in 114 (68.7%) patients, follow-up of baseline WMH in 19 (11.4%), follow-up of a single small unruptured aneurysm in 10 (6.0%), and follow-up of an old lacunar infarction in 23 (13.9%, **S1 Table, panel B**). The design of this study was reviewed and approved by the institutional review board of Seoul National University Hospital. As patient information was anonymized and de-identified prior to our analysis, the requirement for informed consent was waived.

### Acquisition of clinical data

Demographic information and clinical profiles, including the patients’ age; sex; body mass index (BMI, kg/m^2^)[30]’ and presence of hypertension, diabetes mellitus, hyperlipidemia, coronary heart disease, stroke history, and smoking habits in the last five years were evaluated. Regular use of medications during the follow-up period including statins, antithrombotic agents, and antihypertensive mediations including angiotensin-converting enzyme inhibitors, aldosterone-receptor blockers, and calcium channel blockers were also obtained from the medical records.[10] Systolic blood pressure (SBP, mmHg) and diastolic blood pressure (DBP, mmHg) were obtained using an electronic manometer after more than 10 minutes of rest in the sitting position. Pulse pressure (PP, mmHg) was defined as SBP-DBP. Changes in blood pressure values and BMI between the baseline and at the end of the follow-up period were also calculated, and designated as ΔSBP, ΔDBP, ΔPP, and ΔBMI, respectively.

### Laboratory measurements

Serum cystatin C was measured from fasting blood samples by means of a particle-enhanced immunonephelometric assay (N Latex Cystatin C, Siemens Healthcare Diagnostics, Inc., Tarrytown, NY, USA) using a BN II nephelometer (Siemens Healthcare Diagnostics, Inc., Tarrytown, NY, USA).[19] GFR was estimated using serum creatinine and cystatin C levels, per the Chronic Kidney Disease-Epidemiology Collaboration (CKD-EPI) equation.[20] The urine spot microalbumin/creatinine ratio (microgram/milligram creatinine) was measured using nephelometry, with a ratio ≥30 mg/g indicative of the presence of microalbuminuria.[19]

Other laboratory parameters for cerebrovascular risk factors, including total cholesterol (TC, mg/dL), low-density lipoprotein (LDL, mg/dL) cholesterol, hemoglobin A1c (HbA1c, %), and the inflammation marker C-reactive protein (CRP, mg/dL), were also measured at baseline and the end of follow-up.[30] Changes in these parameters between the baseline and the end of follow-up were also calculated, and designated as ΔTC, ΔLDL, ΔHbA1c, and ΔCRP.

### Magnetic resonance imaging and volumetric analysis

MRI was performed using a 1.5-T imaging unit with an 8-channel head coil (Philips Ingenia; Philips, Best, Netherlands) under protocols that commonly included axial T1-/T2-weighted images, gradient echo (GRE) images, fluid-attenuated inversion recovery (FLAIR) sequences, intracranial time-of-flight (TOF) angiography, and a contrast-enhanced MRA. FLAIR sequences were obtained with the following parameters: slice thickness/gap of 4.0/0.0 mm, 24–27 slices covering the entire brain, repetition time/echo time (TR/TE) = 9000–9900/97–163 ms, a field-of-view (FOV) = 240 × 240 mm, and matrix = 220 × 220. FLAIR and MRA were reviewed to evaluate the presence or mechanism of preexisting ischemic lesions, GRE to identify preexisting intracerebral hemorrhage, and TOF and contrast-enhanced MRA to exclude subjects with ≥30% stenosis in the intracranial/extracranial arteries.[31,32] The MRI protocols used were identical between the baseline and follow-up MRI evaluations. Images were reviewed by a radiologist (YJR, 6 years of experience), who was blinded to all patient data.

For the quantitative analysis of the WMH volume, the two-dimensional FLAIR images were registered in an offline workstation. WMH was defined as the observation of hyper-intensity in the white matter area.[1–3] Areas of old infarction, which had clean or sharp edges and which appeared as relatively dark signals on FLAIR images, were excluded from the measurement of WMH. WMH lesions were outlined by a neurologist (WJL, five years of experience), using NeuRoi (Nottingham university, Nottingham, UK), a semi-automated freeware that has been used in previous studies, [17,33] blinded to all patient information and as to whether the images were a baseline or a follow-up image. Cases of WMH identified in the brainstem or in the cerebellum were excluded. The brain volume and the total WMH lesion volume were also measured using the NeuRoi software.[17,33] To evaluate the intra-rater reliability of the volumetric analysis, 20 MRI scans were randomly allocated for repeated measurements. Intra-class correlation coefficients for WMH volumes were (0.98, 95% confidence interval [CI]: 0.97–0.99). The change in WMH volume was calculated by subtracting the lesion volume at the time of the baseline MRI from the lesion volume at the time of the follow-up MRI. To adjust for the effect of heterogeneous intervals of MRI evaluations, WMH progression rate was defined as the change in WMH volume divided by the MRI intervals (mL/year). Full clinical, laboratory, and radiologic data are available in the supplemental S1 Dataset.

### Statistical analysis

Data were reported as a number (percentage), mean±standard deviation, or a median (interquartile range, IQR). For univariate analysis, Pearson’s correlation analysis was applied to measure the correlations between continuous variables and WMH progression rate. For categorical variables, mean WMH progression rates were compared between each subgroup using Student’s *t*-tests or the Mann–Whitney *U* test. Variables with *P* values <0.15 in univariate analyses were entered into a multivariate linear regression analysis using an enter method.

In the multivariate linear regression analysis, CRP was log-transformed to obtain a normal distribution, as the distribution of the CRP level was significantly skewed. Other continuous variables such as the baseline WMH volume, cystatin C, GFR, TC, LDL, HbA1c, SBP, DBP, PP, BMI, and their Δ values were normally distributed. As three outlier values (> 3 standard deviation) were observed in the WMH progression rate, they were excluded from the linear regression analysis. After the multiple linear regression model was obtained, a scatterplot of the standardized predicted values and one of the standardized residuals was drawn to check the assumption for linearity. To examine the assumption for the normal distribution, a histogram of the standardized residuals and a normal probability (P-P) plot of the standardized residuals were drawn. To access the multicollinearity between variables, the variance inflation factor (VIF) was measured, where a value of >3.00 indicates a significant collinearity. *P* values <0.05 were noted as statistically significant for every analysis. SPSS 22.0 (IBM Corp., Armonk, NY, USA) was used for all statistical analyses.

## Results

**Fig 1:**
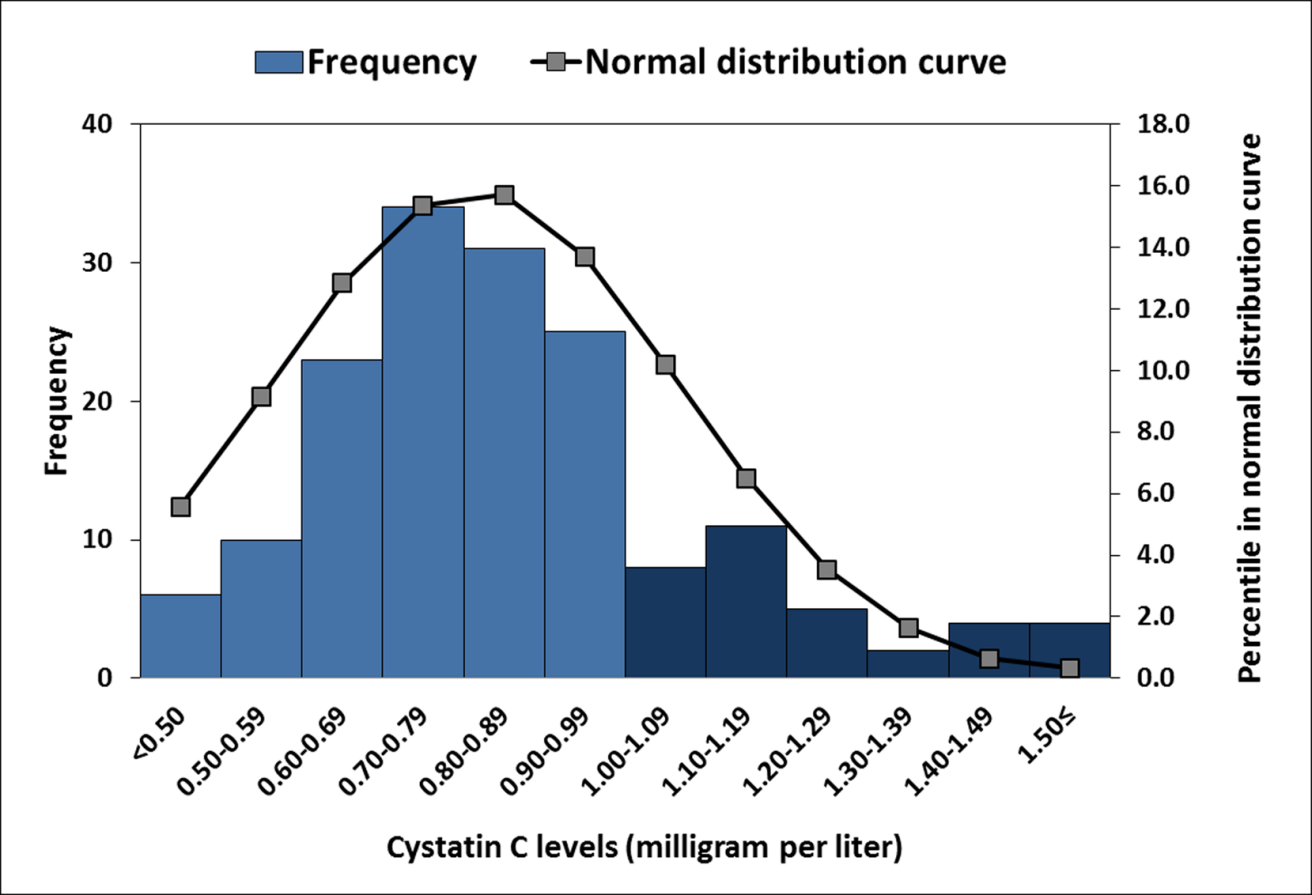
Distribution of the study population according to cystatin C values. Bar graphs denote the number of patients in each subgroup defined by intervals of cystatin C value. Sixty (24.2%) patients had a cystatin C level higher than 1.00 mg/L (dark blue bars). A normal distribution curve was also demonstrated.

Among the 166 individuals (94 [56.6%] men, mean age: 66.5±8.2 years, range: 50−87 years), the baseline WMH volume was 9.61±13.17 ml, GFR was 77.3±22.8 ml/min, and microalbuminuria was present in 50 (30.1%) patients. The mean baseline cystatin C level was 0.92±0.52 mg/L, and 34 (20.5%) patients had a cystatin C level of ≥1.00 mg/L (**Fig 1**). At the end of follow-up, laboratory evaluations performed at an average of 38.1±3.4 months after the initial laboratory evaluations, and revealed that the mean ΔGFR was 2.4±7.9 mL/min and the mean Δcystatin C was 0.03±0.34 mg/L. The follow-up MRI evaluations were performed at an average of 37.9±3.4 months (range: 34−45 months) after the initial MRI. The observed change in WMH volume was 3.64±6.85 ml, and the WMH progression rate was 1.18±2.28 ml/year (**Table 1**).

**Table 1:** Clinical, laboratory, and white matter hyperintensity profiles of the study population.

Clinical profiles	N = 166	Medication profiles	
Age, years	66.5 (8.2)	Statin	114 (68.1)
Male sex	94 (56.6)	Antithrombotic agents	149 (89.8)
Previous lacunar stroke	59 (35.5)	Antihypertensive medication	103 (62.0)
Hypertension	107 (64.5)	**Radiographic profiles**	
Diabetes mellitus	63 (38.0)	Baseline WMH volume, mL	9.61 (13.17)
Hyperlipidemia	68 (41.0)	Median (IQR)	5.27 (1.80–13.92)
Coronary heart disease	25 (15.1)	MRI interval, months	37.9 (3.4)
Smoking in past 5 years	24 (14.5)	ΔWMH volume, mL	3.64 (6.85)
BMI, (kg/m^2^)	23.7 (2.8)	Median (IQR)	0.90 (0.03–4.22)
ΔBMI, (kg/m^2^)	0.1 (1.1)	WMH volume progression rate, mL/year	1.18 (2.28)
		Median (IQR)	0.27 (0.01–1.28)
**Laboratory profiles**		**Follow-up laboratory profiles**	
SBP, mmHg	138.5 (24.5)	ΔSBP, mmHg	-2.3 (7.9)
DBP, mmHg	79.6 (11.6)	ΔDBP, mmHg	-0.3 (4.4)
PP, mmHg	58.9 (17.3)	ΔPP, mmHg	-2.0 (5.2)
GFR, mL/min	77.3 (22.8)	ΔGFR, mL/min	2.4 (7.9)
TC (mg/dL)	157.8 (33.9)	ΔTC (mg/dL)	0.4 (31.1)
LDL (mg/dL)	86.3 (27.8)	ΔLDL (mg/dL)	1.9 (22.8)
HbA1c (%)	6.2 (1.0)	ΔHbA1c (%)	0.0 (0.6)
CRP (mg/dL)	0.54 (1.13)	ΔCRP (mg/dL)	-0.12 (0.78)
Cystatin C (mg/L)	0.92 (0.52)	ΔCystatin C (mg/L)	0.03 (0.34)
Mircoalbuminuria	50 (30.1)	Laboratory interval, months	38.1 (3.4)

Data are reported as number (percentage), as mean± standard deviation, or as median (interquartile range, IQR). BMI: body mass index, SBP systolic blood pressure, DBP: diastolic blood pressure, PP: pulse pressure, GFR: glomerular filtration rate, TC: total cholesterol, LDL: low-density lipoprotein, HbA1c: hemoglobin A1c, CRP: C-reactive protein, WMH: white matter hyperintensity.

Correlation analyses of the continuous variables revealed that the progression rate of WMH volume was significantly associated with the baseline WMH volume (*P*<0.001), patient age (*P*<0.001), cystatin C level (*P*<0.001), GFR (*P* = 0.047), CRP (*P* = 0.001), and Δcystatin C (*P* = 0.004, **Table 2**). Among the categorical variables including the cerebrovascular risk factors, microalbuminuria, medication profiles, and the indications for the initial MRI evaluations, no statistically significant association was found with WMH progression rate (**Table 3**).

**Table 2:** Correlations co-efficiencies of white-matter hyperintensity progression rate with continuous variables.

	The progression rate of WMH volume (mL/year)	*P*
Baseline WMH volume (mL)	0.450	<0.001**
Age	0.294	<0.001**
Cystatin C (mg/L)	0.279	<0.001**
GFR, mL/min	-0.154	0.047*
BMI, (kg/m^2^)	-0.114	0.145
SBP, mmHg	0.031	0.691
DBP, mmHg	-0.022	0.780
PP, mmHg	0.059	0.453
TC (mg/dL)	-0.039	0.615
LDL (mg/dL)	0.018	0.821
HbA1c (%)	0.100	0.200
CRP (mg/dL)	0.263	0.001**
ΔCystatin C (mg/L)	-0.225	0.004**
ΔGFR, mL/min	0.091	0.243
ΔBMI, (kg/m^2^)	0.005	0.951
ΔSBP, mmHg	-0.018	0.822
ΔDBP, mmHg	0.038	0.626
ΔPP, mmHg	-0.059	0.453
ΔTC (mg/dL)	-0.090	0.248
ΔLDL (mg/dL)	-0.030	0.699
ΔHbA1c (%)	0.054	0.488
ΔCRP (mg/dL)	-0.132	0.091

WMH: white matter hyperintensity, GFR: glomerular filtration rate, BMI: body mass index, SBP systolic blood pressure, DBP: diastolic blood pressure, PP: pulse pressure, TC: total cholesterol, LDL: low-density lipoprotein, HbA1c: hemoglobin A1c, CRP: C-reactive protein

*P<0.05

**P<0.01.

**Table 3:** Mean and standard deviations of the white-matter hyperintensity progression rate in the patients grouped per the categorical parameters.

N = 166	WMH progression rate, mL/year			
		Yes	No	*P*
**Clinical parameters**				
Male sex		1.09 (1.79)	1.31 (2.81)	0.537
Previous lacunar stroke		1.31 (1.88)	1.12 (2.49)	0.599
Hypertension		1.37 (2.3)	0.85 (2.24)	0.157
Diabetes mellitus		1.52 (2.49)	0.98 (2.13)	0.136
Hyperlipidemia		1.56 (2.86)	0.92 (1.75)	0.104
Coronary heart disease		1.22 (2.37)	1.18 (2.28)	0.927
Smoking in past 5 years		1.76 (2.49)	1.09 (2.24)	0.181
Mircoalbuminuria		1.14 (1.76)	1.21 (2.48)	0.859
Statin		1.33 (2.4)	0.87 (2.02)	0.229
Antithrombotic agents		1.12 (2.08)	1.79 (3.67)	0.469
Antihypertensive medication		1.3 (2.18)	0.99 (2.46)	0.396
**Indications for the initial MRI/MRA evaluation**				
Medical check-up program		0.91 (1.55)	1.10 (1.96)	0.517
Headache or dizziness		0.83 (1.74)	1.03 (1.71)	0.530
Intracranial small aneurysm		1.58 (2.08)	0.95 (1.69)	0.261
Old lacunar infarction		1.43 (2.35)	0.94 (1.64)	0.421

Data are reported as mean (standard deviation).

In a multivariate linear regression analysis that adjusted for the total brain volume, cystatin C level was significantly associated with WMH progression rate (B coefficient = 0.856; 95% confidence interval [CI] 0.174−1.538; *P* = 0.014), along with the baseline WMH volume (B = 0.039; 95% CI 0.019−0.059; *P*<0.001, **Table 4**). However, age, GFR, BMI, the log value of CRP, Δcystatin C, ΔCRP, and vascular risk factors were not significantly associated with WMH progression rate. In the scatterplot of the standardized predicted values and the standardized residuals, a random and even distribution of the standardized residuals around the zero line was observed. The standardized residual was normally distributed in a histogram, and the P-P plot distribution was near the comparison line. VIF values for each variable were <2.0.

**Table 4:** Linear regression analyses for factors associated with white-matter hyperintensity progression rate.

	B (95% CI)	β	P
	-1.583 (-5.700–2.533)		0.448
Baseline WMH volume (mL)	0.039 (0.019–0.059)	0.285	<0.001**
Age	0.027 (-0.004–0.057)	0.126	0.085
Cystatin C (mg/L)	0.856 (0.174–1.538)	0.263	0.014*
GFR, mL/min	0.005 (-0.007–0.017)	0.066	0.418
BMI, (kg/m^2^)	-0.040 (-0.150–0.069)	-0.051	0.468
CRP^†^ (mg/dL)	0.078 (-0.062–0.218)	0.085	0.272
ΔCystatin C (mg/L)	-0.580 (-1.507–0.347)	-0.114	0.218
ΔCRP (mg/dL)	0.057 (-0.289–0.402)	0.026	0.747
Diabetes mellitus	0.297 (-0.183–0.778)	0.084	0.223
Hyperlipidemia	0.364 (-0.112–0.841)	0.104	0.133
Total brain volume (mL)	0.000 (0.000–0.001)	0.088	0.193

R^2^ = 0.366 and *P*<0.001 for the linear regression equation.

B: unstandardized coefficient, β: standardized coefficient, CI: confidence interval, WMH: white matter hyperintensity, GFR: glomerular filtration rate, BMI: body mass index, CRP: C-reactive protein.

^†^The variables were log-transformed to obtain a normal distribution

*P<0.05

**P<0.01.

**Fig 2:**
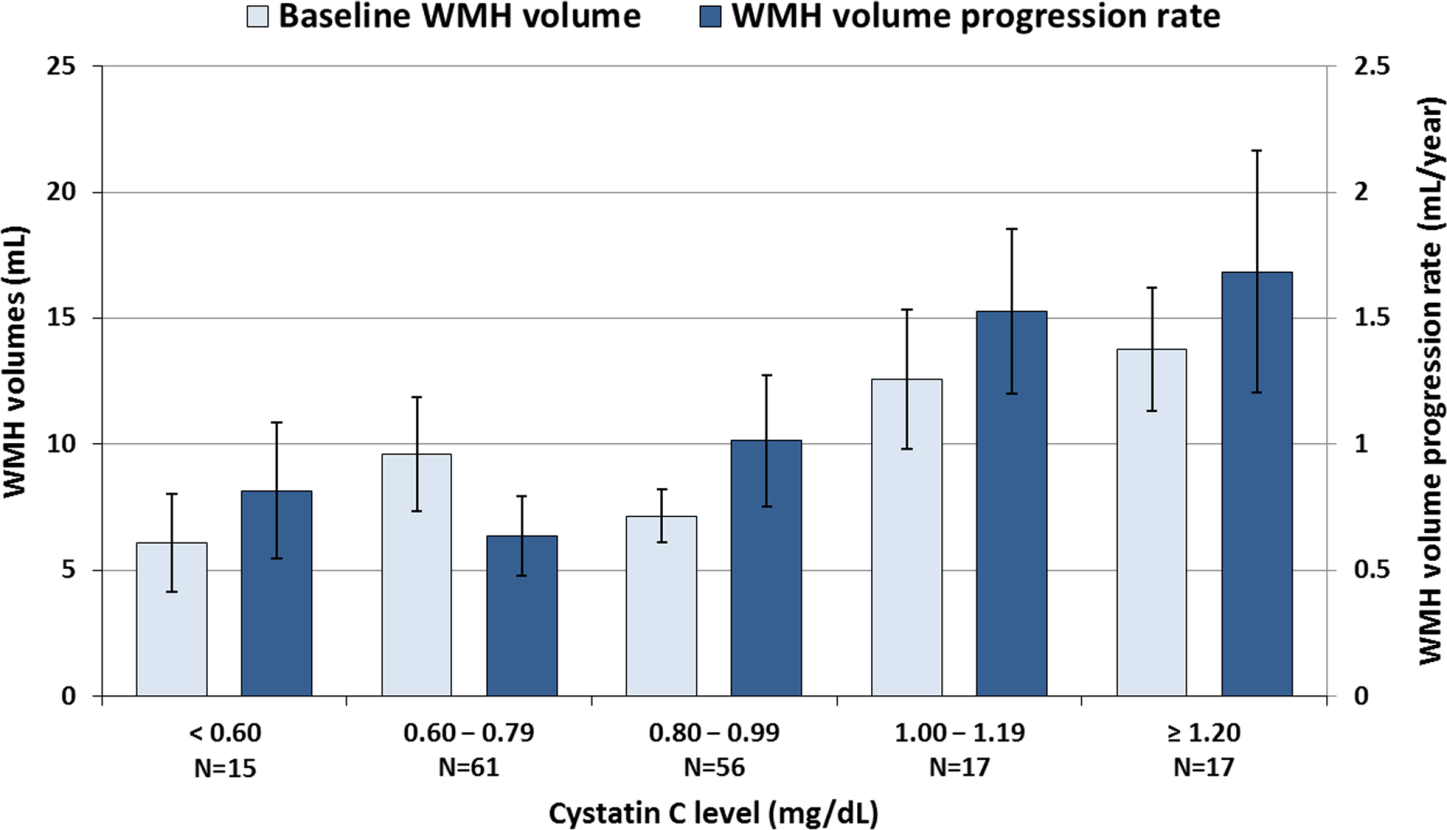
Profiles of baseline white matter hyperintensity (WMH) volume and the change of WMH volume at follow-up, according to cystatin C levels. Horizontal lines above the bars denote standard errors. WMH: white matter hyperintensity.

When the mean WMH volumes of the patients of the subgroups divided according to cystatin C levels were calculated, patients with higher cystatin C levels were significantly correlated with having a higher progression rate of WMH, but not with a higher baseline WMH volume (*P* = 0.014 and *P* = 0.118 for linear trends, respectively). Furthermore, patients with a baseline level of cystatin C ≥1.00 mg/L exhibited a much higher progression rate of WMH volume, as compared with those with a baseline level of cystatin C <1.00 mg/L (1.60±1.91 mL/year vs. 0.82±1.63 mL/year, *P* = 0.010), suggesting that a level of cystatin C ≥1.00 mg/L might be an indicator for an increased risk of WMH progression (**Fig 2**).

## Discussion

In the present study, we observed a linear relationship between the cystatin C level and the progression of cerebral WMH, which was independent of the creatinine-cystatin C based GFR. Notably, this association remained valid after adjusting for previously established predicting factors of WMH progression, including increasing patient age, systolic and pulse blood pressure,[1,8,11] use of antihypertensive medications,[10] and microalbuminuria.[19] These findings suggest that cystatin C may have more direct associations with the underlying pathological mechanisms of WMH progression than as a precise marker of GFR. Moreover, a certain cystatin C level (≥1.00 mg/L) was associated with a higher progression rate of WMH, implying that this cystatin C level could be used as an indicator for an increased risk of WMH progression.

Numerous studies have reported that decreased kidney function is associated with cerebral WMH.[14–16] Although not fully elucidated, the pathophysiologic base of this relation is assumed to be the noted similarities between the glomerular and cerebral microvascular systems, which are both comprised of abundant arteriolar beds with high compliance, and are thus susceptible to aging-related increments in the stiffness of the arterioles and endothelial dysfunction.[34] Similarly, previous cross-sectional studies have indicated that the serum level of cystatin C, as a reliable marker for kidney function, is associated with various cerebrovascular complications.[19,21,35] However, the current study suggests that cystatin C may have a more direct pathophysiologic relation with the progression of WMH than as a kidney function marker, because its association with WMH progression rate was independent from the GFR.

The mechanism of solute clearance in CNS via the glymphatic system might explain how the cystatin C level might reflect the pathomechanism of cerebral WMH progression. First, given that an increased cystatin C level specifically correlates with a reduction in small-artery elasticity,[36] cystatin C level might represent the decreased pulsatility of the cerebral penetrating arterioles, which induces the impairment of glymphatic solute clearance and subsequent accumulation of toxic metabolites and swelling in brain parenchyma.[4,22,37] Second, cystatin C may reflect the activity of the neuronal degeneration process in the brain. Cystatin C is secreted from neurons, astrocytes, and microglia; is highly concentrated in brain; and its level in the brain parenchyma increases in response to the degeneration of neurons.[19,24,25] Third, cystatin C also accumulates in the smooth muscles of the cerebral penetrating arterioles.[22,23] This finding is widely observed in patients with cerebral-amyloid angiopathy, Alzheimer’s dementia, and even cognitively normal aged individuals.[24,25] This might result from the reduced pulsation of the penetrating arterioles and impaired drainage of the solutes to the downstream glymphatic system. Moreover, locally concentrated cystatin C in vessel walls facilitates the dysregulation of the composition of the basement membrane, and the disruption of smooth muscle layer, by inducing an imbalance between proteases and their inhibitors.[22–25,37]

The primary limitation of the present study is that the intervals between the initial and follow-up MRI scans were not standardized, due to the study's retrospective design. This issue might be resolved in future prospective studies by applying a standardized evaluation protocol. Additionally, *in vivo* studies correlating cerebrovascular or parenchymal cystatin C deposition with microvascular or neuronal structural integrity should be performed in order to further elucidate the CNS-specific pathomechanistic role of cystatin C in the progression of cerebral WMH.

## Conclusion

Along with the baseline WMH degree of severity, cystatin C levels are associated with long-term progression of cerebral WMH, independently of the creatinine-based GFR. Serum cystatin C level might be a marker for the long-term progression of cerebral WMH.

## Supporting information

S1 Table Descriptions of excluded patients and indications of initial and follow-up evaluations. (DOCX)

S1 Dataset Full dataset of the study population. (XLSX)
