# Supplementary material for: Cystatin C, a potential marker for cerebral microvascular compliance, is associated with white-matter hyperintensities progression
Source: PLoS One. 2017 Sep 14;12(9):e0184999. doi: 10.1371/journal.pone.0184999 (PMC5599063; doi:10.1371/journal.pone.0184999)
Supplement: S1 Table — (DOCX) [file pone.0184999.s001.docx]

Online Supplemental Data

**Cystatin C, a potential marker for cerebral microvascular compliance, is associated with white-matter hyperintensities progression**

**List of the supplements:** 1 Supplemental Table and 1 Supplemental Dataset

**Supplemental S1 Table.** Descriptions of excluded patients and indications of initial and follow-up evaluations

**Supplemental S1 Dataset.** Full dataset of the study population (Excel file)

**Supplemental S1 Table .** Descriptions of excluded patients and indications of initial and follow-up evaluations

| 1. Inclusion Criteria^†^ |  | N=263 |
| --- | --- | --- |
| 1. ≥50 years of age |  |  |
| 1. Baseline brain MRI/MRA and laboratory evaluation including serum cystatin C levels |  |  |
| 1. Follow-up MRI/MRA and laboratory evaluation including serum cystatin C levels within 34–45 months of the initial evaluation |  |  |
| Exclusion criteria | Excluded patients^‡^ | Remaining patients |
| 1. A ≥30% stenosis of the intra/extracranial ICA or MCAs | 35 (13.3%) | 228 |
| 1. Active systemic illness or unable to carry out daily activities independently ^a^ | 10 (4.4%) | 218 |
| 1. A stroke from etiologies other than a lacunar infarction, (≤90 days) lacunar stroke, or a major head trauma ^b^ | 49 (22.5%) | 169 |
| 1. CNS inflammatory/degenerative disorder ^c^ | 3 (1.8%) | 166 |
| 1. A poor MRI image quality | 0 (0.0%) | 166 |
| 1. Indications for initial MRI/MRA |  | Number (%) |
| 1. Medical check-up program ^d^ | | 100 (60.2%) |
| 1. Evaluation for headache or dizziness ^e^ | | 37 (22.3%) |
| 1. Follow-up of a single small (≤3mm) un-ruptured intracranial aneurysm in locations other than distal ICA or MCA | | 10 (6.0%) |
| 1. Follow-up of an old lacunar infarction | | 19 (11.4%) |
| Indications for follow-up MRI/MRA | |  |
| 1. Medical check-up program ^f^ | | 114 (68.7%) |
| 1. Follow-up of baseline WMH ^g^ | | 19 (11.4%) |
| 1. Follow-up of single small (≤3mm) un-ruptured intracranial aneurysm | | 10 (6.0%) |
| 1. Follow-up of an old lacunar infarction ^h^ | | 23 (13.9%) |

MRI/MRA: magnetic resonance image/angiography, ICA: internal carotid artery, MCA: middle cerebral artery, WMH: white matter hyperintensity, ^†^ all consecutive patients who visited a tertiary hospital between January 2005 and March 2012, ^‡^ percentages are the proportion of patients excluded by the relevant criterion. ^a^ A prior history or interval development of: cancer in six patients, end stage renal disease in three patients, and transplantation of organs in one patient. ^b^ Strokes from etiologies other than a lacunar infarction in 42 patients, a (≤90 days) lacunar stroke in 6 patients, and a major head trauma in one patient. ^c^ Includes CNS autoimmune diseases, history of CNS infections, vasculopathies involving CNS, and Alzheimer’s dementia, ^d^ Medical check-up program provided by Seoul National University Hospital Healthcare System for the aged population, ^e^ Primary or nonspecific headache in 23 patients and dizziness without a CNS origin in 14 patients, ^f^ In this indication, 82 patients performed initial MRI/MRA as a medical check-up program and remaining 32 patients performed initial MRI/MRA for nonspecific headache or dizziness, ^g^ Indications for the initial MRI/TCD evaluations for these patients were a medical check-up program in fourteen patients and for nonspecific headache or dizziness in five patients, ^h^ Four patients in this group were diagnosed as an old lacunar infarction at initial MRI evaluation performed as a medical checkup or during follow-up.
